# Combined HIF-1α blockade and CHIR99021 treatment reverses pulmonary fibrosis via modulation endothelial-to-mesenchymal transition

**DOI:** 10.1016/j.isci.2026.114722

**Published:** 2026-01-22

**Authors:** Jae-Kyung Nam, Seo-Hyun Choi, Ji-Hee Kim, Kyu Jin Choi, Hae-June Lee, Jeeyong Lee, Haeng Ran Seo, Yoon-Jin Lee

## Main text

(iScience *28*, 114028; December 19, 2025)

In the originally published version of this article, the image intended as Figure 4F was mistakenly uploaded as Figure 5D during the final production process. As a result, the figure panel and its corresponding description in the figure legend were incorrectly assigned between Figures 4 and 5 in the published version. Additionally, the legends for Figures 4C and 4E were inadvertently swapped. These errors have now been corrected in the article online. The authors apologize for this oversight and confirm that these changes do not affect the scientific message, results, or conclusions of the article.

**Figure:**
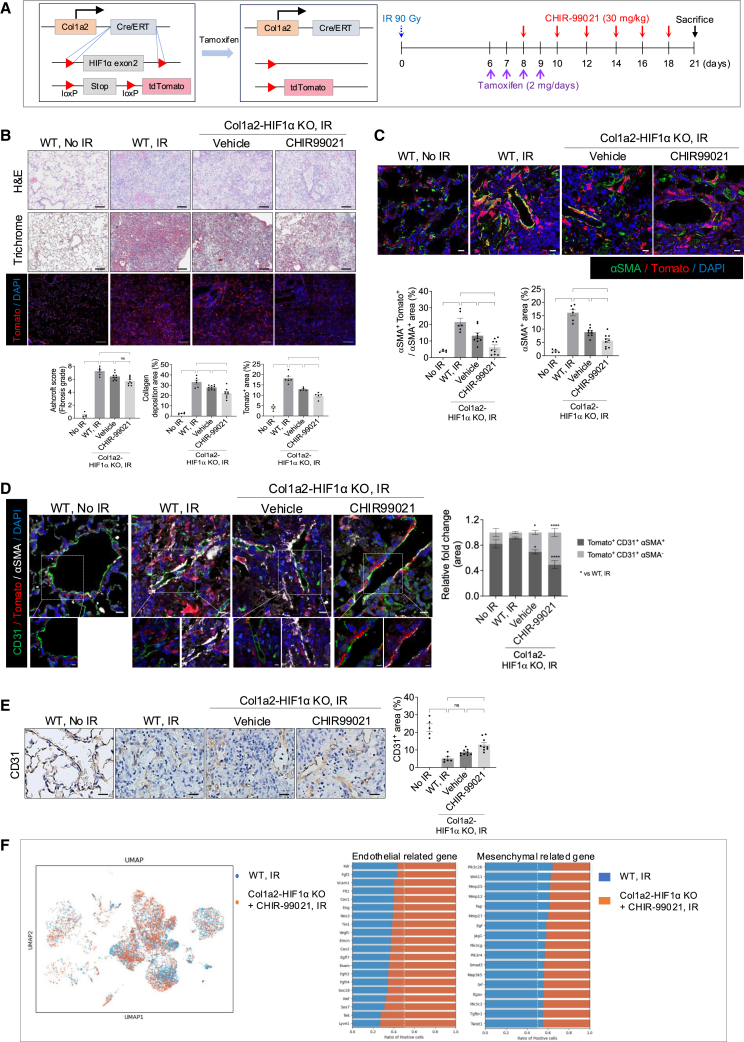
Figure 4. HIF-1α deletion and CHIR99021 synergistically reduce radiation-induced fibrosis and promote endothelial repair (corrected) (A) Schematic representation of collagen irradiation and drug administration in WT and Col1a2-HIF1α KO mice. Col1a2 Cre-ER mutant mice carry a tamoxifen-inducible Cre recombinase. For Tomato expression and HIF1α deletion in fibroblasts, mice carrying a loxp site in the HIF1a gene side and mice carrying the Tomato gene were crossed with Col1a2 Cre-ER mice. Tamoxifen was injected into mice to induce expression of Tomato and deletion of HIF1α in fibroblasts. WT and Col1a2-HIF1α KO mice were irradiated in the left lung with 90 Gy using a 4 mm diameter field. Col1a2-Tomato mice were treated with tamoxifen (2 mg/day) once daily for 4 days starting 6 days post-irradiation and administered CHIR99021 (30 mg/kg) starting 4 days post-irradiation, with dosing continued every 2 days. Lung samples (n ≧ 5/group) were obtained 21 days post-irradiation from non-irradiated and irradiated mice. (B) Representative images of Hematoxylin & eosin staining, Masson trichrome staining and Tomato immunofluorescence staining in non-irradiated or irradiated lung tissues from WT and Col1a2-HIF1α KO mice, with or without drug treatment (magnification, 200×). Scale bar = 40 μm. Scoring of fibrosis grade, quantification of collagen deposition and Tomato^+^ area are shown in the graph. (C) Immunofluorescence staining of Tomato (red) and αSMA (green in the lung tissues of mice 21 days post-irradiation (magnification, 400×). Scale bar = 10 μm. Bar graphs quantify the αSMA and αSMA^+^ Tomato^+^ / αSMA^+^ area. (D) Immunofluorescence staining of CD31(green), Tomato (red) and αSMA (white) in the lung tissues of mice 21 days post-irradiation (magnification, 400×). Scale bar = 10 μm. scale bar of cropped images = 5 μm. Quantification of the Tomato^+^ CD31^+^ αSMA^+^ area and the Tomato^+^ CD31^+^ αSMA^-^ area. (E) Immunohistochemistry staining of CD31 in the lung tissues of mice 21 days post-irradiation (magnification, 200×). scale bar = 10 µm. Bar graphs quantify the CD31 area. (F) Uniform Manifold Approximation and Projection (UMAP) plot of Single-Cell Transcriptomes. The plot was generated with single-cell RNA sequencing data using scanpy package. Each sample is represented by a distinct color: ‘WT, IR’ in blue and ‘Col1a2-HIF1α KO+ CHIR99021, IR’ in orange (left panel). Comparison of endothelial- and mesenchymal-related gene expression between two conditions: ‘WT, IR’ and ‘Col1a2-HIF1α KO + CHIR99021, IR’. The bar plot represents the ratio of positive cells expressing each gene. The y-axis lists genes associated with endothelium or mesenchyme, while the x-axis represents the ratio of positive cells between the two conditions (right panel). In the Ashcroft score graph (A) error bars indicate SD. In all other graphs, error bars indicate SEM. ∗*P* < 0.05, ∗∗*P* < 0.01, ∗∗∗*P* < 0.001 and ∗∗∗∗*P* < 0.0001; ns: not significant (one-way ANOVA for multiple comparisons).

**Figure:**
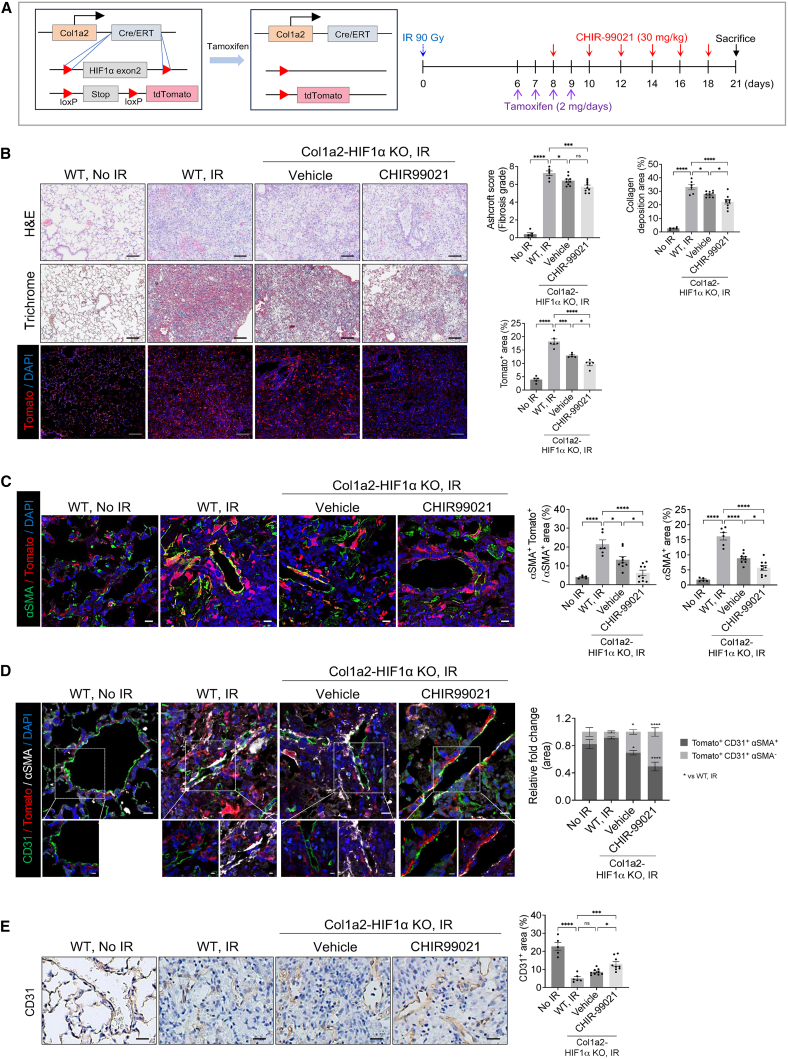
Figure 4. HIF-1α deletion and CHIR99021 synergistically reduce radiation-induced fibrosis and promote endothelial repair (original)

**Figure:**
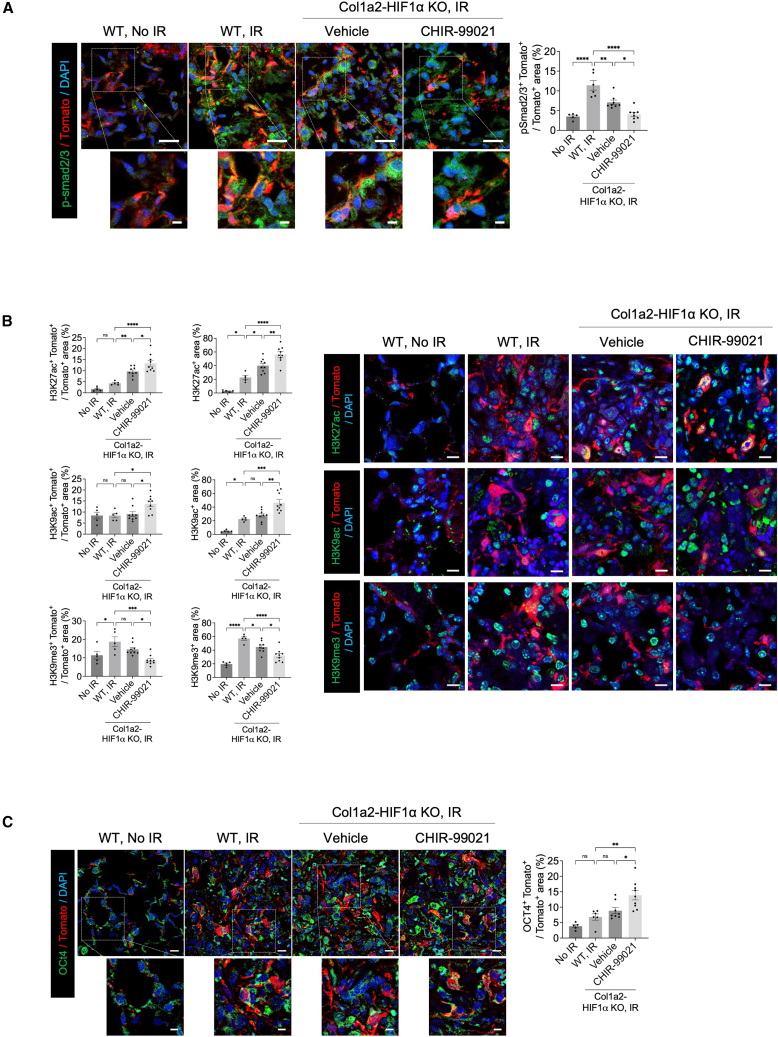
Figure 5. HIF-1α deletion and CHIR99021 synergistically regulate *p*-smad2/3, histone modifications, and pluripotent factor (corrected) (A) Immunofluorescence staining of p-smad2/3 (green) and Tomato (red) in non-irradiated or irradiated lung tissues from WT and Col1a2-HIF1α KO mice 21 days post-irradiation (magnification, 400×). Scale bar = 20 μm. scale bar of cropped images = 5 μm. Quantification of the p-smad2/3^+^Tomato^+^ / Tomato^+^ area. (B) Immunofluorescence staining of histone modifications in lung tissues. Representative images of H3K27ac (green), H3K9ac (green) H3K9me3 (green) and Tomato(red) in the lung tissues of 21 days post-irradiation, treated with or without drug treatment (magnification, 400×) (right panel). Scale bar = 10 μm. Bar graphs quantify the H3K27ac, H3K9ac and H3K9me3 area per nucleus area (left panel). (C) Immunofluorescence staining of OCT4 (green) and Tomato (red) in the lung tissues of mice 21 days post-irradiation (magnification, 400×). Scale bar = 10 μm. scale bar of cropped images = 5 μm. Quantification of the OCT4^+^Tomato^+^ / Tomato^+^ are. All graph error bars indicate SEM. ∗*P* < 0.05, ∗∗*P* < 0.01, ∗∗∗*P* < 0.001 and ∗∗∗∗*P* < 0.0001; ns: not significant (one-way ANOVA for multiple comparisons).

**Figure:**
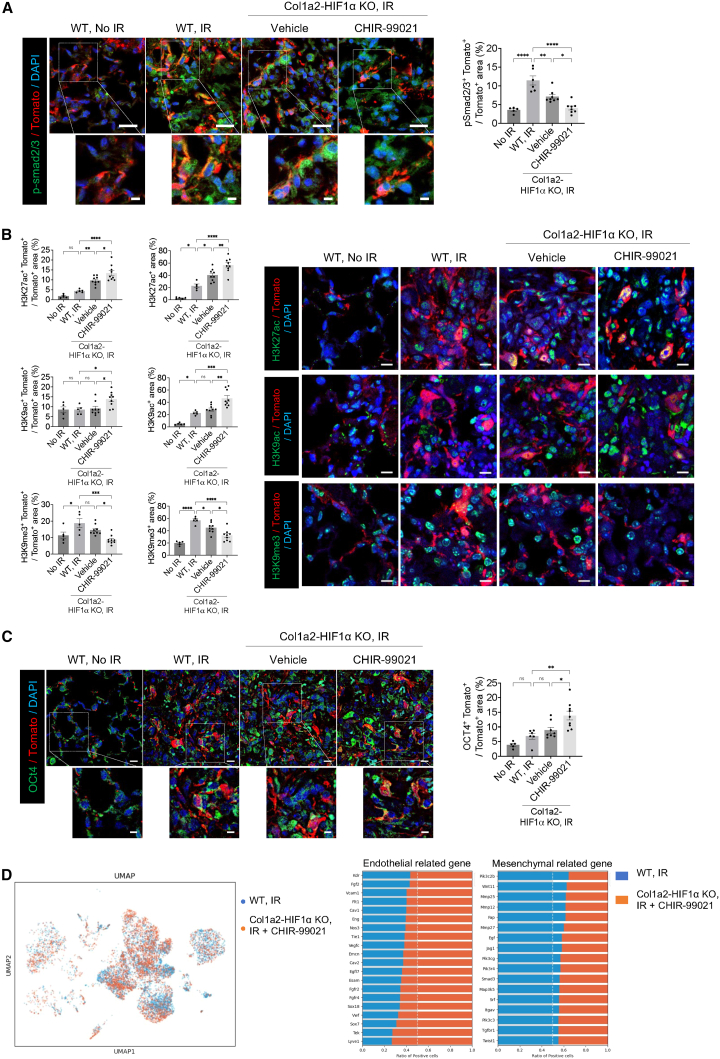
Figure 5. HIF-1α deletion and CHIR99021 synergistically regulate *p*-smad2/3, histone modifications, and pluripotent factor (original)

